# Hypertension Accompanied by Hyperaldosteronism, Hyperkalemia, and Hyperchloremic Acidosis: A Case Report and Literature Review

**DOI:** 10.1155/2020/1635413

**Published:** 2020-07-23

**Authors:** Yunyun Yang, Yang Ou, Yan Ren, Haoming Tian, Tao Chen

**Affiliations:** Department of Endocrinology and Metabolism, Adrenal Center, West China Hospital of Sichuan University, Chengdu, Sichuan 610041, China

## Abstract

This study reported on a 24-year-old woman who complained of a paroxysmal headache for six months and elevated blood pressure for four months. Laboratory examination revealed increased serum potassium and chloride levels, metabolic acidosis, suppressed renin activity, and increased plasma aldosterone concentration. Whole-exome sequencing revealed a heterozygous mutation in exon 11 of the KLHL3 gene: c.1298G > A. After treatment with low-dose hydrochlorothiazide, her clinical problems were controlled. This patient is the first case of Gordon syndrome (GS) within the Chinese population caused by a heterozygous KLHL3 mutation. A systematic review of the published literature identified 27 patients with GS caused by a KLHL3 mutation. These patients had a mean age of 28.2 ± 22.0 years; 74.1% presented with hypertension, 76.9% with hyperkalemia, and 59.1% with metabolic acidosis. The patients also had varying levels of plasma renin activity and plasma aldosterone concentrations.

## 1. Introduction

Gordon syndrome (GS), also known as familial hyperkalemic hypertension or pseudohypoaldosteronism type II (PHA II), is a rare type of familial hypertension. GS results from excessive activation of the thiazide-sensitive Na/Cl cotransporter (NCC) in the distal convoluted tubules, which leads to an increase in NaCl reabsorption and decreases in K^+^ and H^+^ excretion, eventually resulting in high blood pressure, hyperkalemia, hyperchloremic metabolic acidosis, suppressed renin levels, and variable aldosterone levels. Other clinical manifestations of GS include growth disorders, myasthenia, periodic paralysis, skeletal abnormalities, and psychomotor delay [1]. Mutations in at least four genes are responsible for GS, including the WNK1, WNK4, KLHL3, and CUL3 genes. A large cohort study of PHA II kindreds illustrated that mutations in KLHL3 and CUL3 were the leading causes (approximately 79%), and mutations in WNK1 or WNK4 only accounted for a small number of PHAII kindreds (approximately 13%).

The present case was a 24-year-old young woman with hypertension, hyperkalemia, hyperchloremia, metabolic acidosis, low renin, and elevated aldosterone. Whole-exome sequencing of the patient revealed a KLHL3 heterozygous mutation: c.1298G > A. After treatment with low-dose hydrochlorothiazide, the patient's high blood pressure, hyperkalemia, and hyperchloremic metabolic acidosis gradually normalized. In addition, the present study systematically reviewed the published literature about the Gordon syndrome with KLHL3 mutations and summarized the clinical characteristics of genetically confirmed GS cases.

## 2. Case Presentation

The patient was a 24-year-old young woman complaining of a paroxysmal headache for six months and elevated blood pressure for four months. Six months ago, she experienced an episodic headache after sleep insufficiency. Four months ago, she was found with hypertension (up to 150/109 mmHg) in a health examination. The patient had regular menstruation. Her father, grandfather, and grandmother had hypertension. Physical examination of this patient showed no abnormal signs except elevated blood pressure. As shown in Table 1, the patient had elevated levels of serum potassium and chloride, decreased arterial blood pH, standard bicarbonate concentration, and normal serum creatinine and glomerular filtration rate. The patient had suppressed plasma renin activity, elevated plasma aldosterone concentration, normal cortisol circadian rhythm and levels, and normal levels of 24-hour urinary free cortisol, adrenocorticotropin, and serum catecholamine. Moreover, the captopril challenge test of the patient suggested that her plasma aldosterone was suppressed by 31.6%. Her sex hormones, thyroid hormone, and parathyroid hormone were normal. Magnetic resonance imaging (MRI) examination suggested no obvious abnormality in the bilateral adrenal glands. Ultrasound of the urinary system indicated uric acid crystals in the right kidney.

The patient's father had hypertension, and her mother's blood pressure was normal; both were unavailable for laboratory examination and genetic screening. Her elder brother had normal blood pressure, normal levels of serum potassium and serum chlorine, and normal arterial blood gas analysis results.

**Figure 1:**
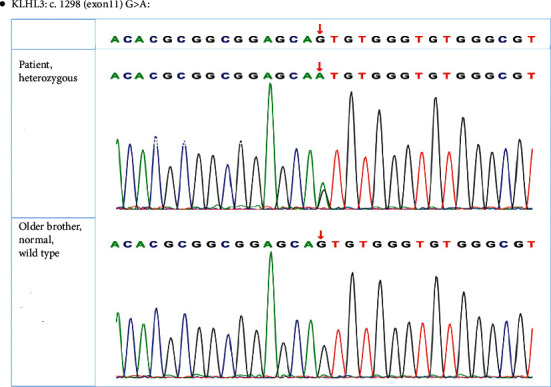
Whole-exome sequencing showing a novel KLHL3 mutation.

The peripheral blood genomic DNA of the patient and her elder brother were extracted (Tiangen DNA extraction kit). Next-generation sequencing was applied to capture suspicious mutation sites in the index patient (Illumina HiSeq 2500 system). The suspected mutation was confirmed by direct sequencing of the index patient and other available individuals (Beijing Zhiyin Oriental Transformational Medicine Research Center Co., Ltd.). The patient was identified with a heterozygous mutation in exon 11 of the KLHL3 gene, c.1298G > A, which led to 433^rd^ amino acid serine to be replaced by asparagine (S433N). Her elder brother was wild type at this site (Figure 1). According to the genetic findings, as well as typical clinical manifestations, the patient was diagnosed with the Gordon syndrome.

After treatment with low-dose hydrochlorothiazide (25 mg/day), her blood pressure fluctuated in the range of 90–100/70–80 mmHg, her blood potassium and chlorine, plasma renin activity, and aldosterone concentration returned to normal, and metabolic acidosis was corrected (Table 1). The patient continued to take the low-dose hydrochlorothiazide treatment after discharge. Her blood pressure and serum potassium were normal three months later at follow-up.

## 3. Review of Clinical Features and Laboratory Findings of GS Patients

To comprehensively review the clinical characteristics of GS caused by the KLHL3 gene mutation, we searched for primary studies published through December 2019 on MEDLINE (PubMed) and the Chinese Biomedical Database (CBM). The MeSH terms “Gordon syndrome,” “Pseudohypoaldosteronism type II,” and “familial hyperkalemic hypertension” were used for the literature search. The retrieved articles in English and Chinese languages were independently screened by two authors (YY Yang and T Chen). Full-text case studies were included only if they had available data on clinical features and laboratory findings. Clinical data, including country of origin, age at symptom onset, gender, and clinical manifestations such as blood pressure, serum potassium, arterial pH value, plasma renin activity (PRA)/concentration (PRC), and plasma aldosterone concentration (PAC), were sorted out and analyzed.

We identified 27 studies in total, then excluded 22 studies that lacked genetic tests or detailed clinical manifestations or that had mutations other than those in the KLHL3 gene, and eventually included five studies that contained 27 affected members from 8 pedigrees (Table 2). No studies in the Chinese language about the KLHL3 gene mutation were retrieved. The mean age at diagnosis was 28.2 ± 22.0 years, and 59.3% of the patients were females. The incidences of hypertension, hyperkalemia, and metabolic acidosis were 74.1%, 76.9%, and 59.1%, respectively. Among GS patients with hypertension (*n* = 20), 45% of them were diagnosed with hypertension before the age of 18 years, and the overall median age was 27.6 years (range: 0.8–62 years). Among GS patients with hyperkalemia (*n* = 20), the median age was 21.6 years. Among GS patients with metabolic acidosis (*n* = 13), the median age was 22.7 years. In addition, 73.9% of GS patients presented with suppressed PRA, 26.1% presented with normal PRA, 38.1% presented with low PAC, 52.4% presented with normal PAC, and 9.5% presented with high PAC.

## 4. Discussion

This study reported on a 24-year-old patient who presented with elevated blood pressure and increased serum potassium and chlorine, as well as decreased arterial blood pH, standard bicarbonate concentration, suppressed PRA, normal serum creatinine level, and normal estimated glomerular filtration rate. The above clinical clues indicated a possible diagnosis of the Gordon syndrome. Whole-exome sequencing revealed that the patient carried a heterozygous mutation in the KLHL3 gene: c.1298G > A, which led to 433^rd^ amino acid serine to be replaced by asparagine (S433N). The patient's elder brother, who had normal blood pressure and normal serum potassium, was wild type at this site.

KLHL3 along with the WNK1, WNK4, and CUL3 genes are involved in the physiologic regulation of NCC transporter activity [1, 7–10]. Mutations in the WNK1, WNK4, KLHL3, and CUL3 genes lead to increased NCC transporter activity and membrane expression [7, 11, 12], enhance the inhibition of ROMK [7, 9, 11], and eventually lead to typical clinical features of GS. Whole-exome sequencing in this patient revealed a heterozygous mutation in exon 11 of KLHL3 (c.1298G > A). This mutation was first reported in a large cohort study of 52 European and American GS kindreds in 2012 [8]. Then, a 2017 study from South Korea reported two cases of GS carrying the same mutation, which included a 25-month-old female infant and her 30-year-old mother. The daughter had hypertension, hyperkalemia (6.9 mmol/L), and metabolic acidosis [13], and the mother had hypertension and hyperkalemia in her twenties [4]. Previously reported GS cases from the Chinese population were mostly caused by WNK4 mutations and CUL3 mutations [14]. To date, no study has reported GS cases with KLHL3 dominant mutations among the Chinese population.

There are significant differences in the severity of clinical phenotypes among GS patients with different gene mutations. CUL3 gene mutation tends to result in much more severe clinical phenotype followed by KLHL3, WNK4, and WNK1 gene mutations. Patients with CUL3 mutations usually developed symptoms at younger ages, with more severe hyperkalemia and metabolic acidosis [7, 8], while patients with KLHL3 mutations usually developed symptoms after adulthood, with mild-to-moderate clinical manifestations.

This patient presented typical features of GS, e.g., hypertension, hyperkalemia, hyperchloremia, and metabolic acidosis, while the systematic review of published studies showed that the clinical manifestations varied among GS patients; hyperkalemia was the most common clinical symptom, which was present in 76.9% of GS patients followed by hypertension (74.1%) and metabolic acidosis (59.1%). Compared with hyperkalemia and metabolic acidosis, hypertension seemed to develop at a later stage (median age: 27.6 versus 21.6 and 22.7 years). This evidence indicated that GS should be suspected when young patients present with hyperkalemia, even those who do not present with hypertension and metabolic acidosis.

Most of the previous studies reported that GS patients had suppressed renin activity but low or normal plasma aldosterone concentrations [2, 4, 14–17]. For instance, one of the South Korean cases of GS caused by the same mutation in the present case (S433N) had a suppressed RAAS (plasma renin activity less than 0.10 ng/mL/h and plasma aldosterone 7.1 ng/dL). Similarly, patients with other sites of KLHL3 mutations (such as Q309R, R528H, H498Y, and L387P) had low plasma renin activity and normal or decreased aldosterone levels [2–5]. The possible mechanism may be associated with increased NCC activity, which leads to increased NaCl reabsorption and volume-dependent hypertension, thus inhibiting the RAAS (decreased renin activity and aldosterone levels).

Interestingly, the present case presented with suppressed PRA and slightly increased PAC. Similarly, as shown in the results of the literature review and other published studies, there were also some cases or pedigrees with GS characterized by an elevated PAC, which was mainly caused by WNK4 or CUL3 mutations [18–20]. The possible mechanism could be related to the regulatory effects of hyperkalemia on aldosterone secretion. For example, one case with a WNK4 mutation had a potassium level of 6.4 mmol/L with a corresponding PAC of 50.7 ng/dL, while when serum potassium decreased to 5.4 mmol/L, the corresponding PAC decreased to 13.7 ng/dL [19]. This phenomenon could also be observed in our patient, whose PAC was 31.0 ng/dL when serum potassium was 6.3 mmol/L, which decreased to 15.2 ng/dL when serum potassium decreased to 4.9 mmol/L. In brief, the above evidence suggested that the RAAS among GS patients is characterized by a suppressed PRA but variable PAC that is positively associated with serum potassium level. Moreover, a suppressed PRA will lead to an increased aldosterone/renin ratio (ARR), which, in addition to elevated blood pressure, might result in inappropriate screening for primary aldosteronism.

GS patients respond to low doses of thiazide diuretics by inhibition of the activity of NCC in the distal nephrons. Previous studies have reported that thiazide diuretics could correct the clinical manifestations, including hypertension, hyperkalemia, and metabolic acidosis, in almost all GS patients [7, 8]. The present patient accepted treatment of low-dose hydrochlorothiazide (25 mg/day). Her elevated blood pressure, serum potassium, and metabolic acidosis were corrected five days later and maintained at normal levels at three months of follow-up.

## 5. Conclusions

This study reported on a case of the Gordon syndrome with a heterozygous mutation (c.1298G > A) in exon 11 of the KLHL3 gene. The patient had typical features of the Gordon syndrome and responded well to low-dose thiazide diuretics. GS patients with KLHL3 gene mutations present with varied clinical manifestations and might be confused with primary aldosteronism.

## Figures and Tables

**Table 1:** Laboratory findings of the index patient.

Biochemical findings	Pre-HCTZ	Post-HCTZ	Reference values
Serum potassium	6.3	4.00	3.5–5.3 mmol/L
Serum sodium	142.8	138.9	137–147 mmol/L
Serum chlorine	114.7	96.4	99–110 mmol/L
Serum creatinine	60	N/A	37–110 *μ*mol/L
eGFR	122	N/A	56–122 mL/min/1.73 m^2^
Arterial blood pH value	7.286	7.400	7.35–7.45
HCO_3_^−^	17.6	26.2	22–27 mmol/L
PRA renin activity	0.14	1.07	0.93–6.56 ng/mL/h
PRC aldosterone	31.00	15.2	9.8–27.6 ng/dl
Serum cortisol (8 AM)	300.2	N/A	147.3–609.3 nmol/L
Serum cortisol (4 PM)	162.0	N/A	34–340 nmol/L
Serum cortisol (midnight)	57.2	N/A	<50 nmol/L
24-h UFC	43.61	N/A	20.3–127.6 *μ*g/24 h
ACTH	21.53	N/A	5.0–78 ng/L

*Note.* eGFR: estimated glomerular filtration rate; ACTH: adrenocorticotropin; UFC: urinary free cortisol; HCO_3_^−^: serum bicarbonate; PRA: plasma renin activity; PRC: plasma aldosterone concentration; and HCTZ: hydrochlorothiazide.

**Table 2:** Systematic review of clinical features and laboratory findings of GS patients with KLHL3 gene mutations.

ID	Mutation	Age (years)	Sex	BP	K^+^	pH	HCO_3_^−^	PRA	PAC
Kelly et al. [2]	H498Y	18	M	150/90	7.3	7.34	18	0.2	19.1

Mitani et al. [3]	L387P	3	M	110/55	6.6	7.34	14.8	0.6	6.6

Park et al. [4]	C164F	0.8	F	115/72	6.3	7.34	19.6	<0.1	6.4
	S433N	1.7	F	110/73	6.9	7.30	12	<0.1	7.1
	S433N	23	F	Hypertension	6.4	NA	NA	NA	NA

Mayan et al. [5]	Q309R	69	F	115/78	5.4	NA	27	2.3	10.6
	Q309R	48	F	147/95	5.2	NA	25.7	0.1	4.1
	Q309R	11	M	104/61	6.4	NA	22.3	0.1	23.8
	Q309R	11	M	108/71	7.2	NA	21.5	0.1	14.0
	Q309R	10	F	103/72	5.3	NA	22.7	0.7	23.9
	R528H	38	F	154/77	5.6	NA	18	3.6	51.5
	R528H	15	F	136/77	6.0	NA	19.7	0.3	4.1
	R528H	13	F	124/77	6.2	NA	19.8	<0.3	14.8
	R528H	8	F	123/78	5.8	NA	19.1	0.7	10.7
	R528H	2	M	146/95	7.0	NA	NA	0.3	28.3

Kliuk et al. [6]	S553L	34	F	158/107	6.2	NA	NA	<0.5	15.5
	S553L	26	M	115/70	7.1	NA	NA	<0.5	6.5
	Q309R	63	M	110/80	5.6	NA	19.0	1.4	4.5
	Q309R	62	F	160/100	4.8	NA	23.6	1.7	8
	Q309R	57	M	120/90	5.1	NA	19.7	NA	16.7
	Q309R	54	M	145/105	6.4	NA	23.1	4.7	24.6
	Q309R	47	F	120/90	5.1	NA	23.8	2.5	21.8
	Q309R	47	F	145/110	5.1	NA	19.5	<1.0	NA
	Q309R	44	M	160/100	NA	NA	NA	NA	NA
	Q309R	18	M	110/70	5.2	NA	23.9	<0.5	NA
	Q309R	15	F	110/80	5.8	NA	20.2	<0.5	NA
	Q309R	12	F	114/76	5.3	NA	22	NA	NA

Present case	S433N	24	F	150/109	6.3	7.286	17.6	0.14	31.0

## Abbreviations

GS: Gordon syndrome
PHA II: Pseudohypoaldosteronism type II
FHH: Familial hyperkalemic hypertension
WNK: With-no-lysine kinase
KLHL3: Kelch-like 3
CUL3: Cullin 3
OSR1: Oxidative stress-responsive gene 1
SPAK: STE20/SPS1-related proline-alanine-rich kinase
CRL: Cullin-RING E3 ubiquitin ligase
DCT: Distal convoluted tubules
NCC: Na/Cl cotransporter
ENaC: Epithelial sodium (Na+) channel
ROMK: Renal outer medullary potassium (K+) channel
RAAS: Renin-angiotensin-aldosterone system
PRA: Plasma renin activity
PAC: Plasma aldosterone concentration
AM: Acidic motif.
